# Beneficial Molecular Adaptations in *BRCA*-Mutation Carriers by Combined HIT/HIRT Intervention: Results from a Pilot Study

**DOI:** 10.3390/cancers12061526

**Published:** 2020-06-10

**Authors:** Daniel A. Bizjak, Sebastian V. W. Schulz, Uwe Schumann, Stephanie Otto, Johannes Kirsten, Florian Ebner, Elena Leinert, Jens Huober, Wolfgang Janni, Jürgen Michael Steinacker

**Affiliations:** 1 Ulm University Hospital, Division of Sports and Rehabilitation Medicine, 89075 Ulm, Germany; uwesd1@gmx.de (U.S.); stephanie.otto@uniklinik-ulm.de (S.O.); johannes.kirsten@uniklinik-ulm.de (J.K.); juergen.steinacker@uniklinik-ulm.de (J.M.S.); 2 Ulm University Hospital, Department of Gynecology and Obstetrics, 89075 Ulm, Germany; florian.ebner@helios-gesundheit.de (F.E.); elena.leinert@uniklinik-ulm.de (E.L.); jens.huober@uniklinik-ulm.de (J.H.); wolfgang.janni@uniklinik-ulm.de (W.J.)

**Keywords:** *BRCA1* mutation, combination HIT/HIRT program, molecular adaptations, BRCA protein/gene expression, anti-oxidative stress/inflammatory response

## Abstract

Based on growing evidence that breast cancer (BRCA) also plays a pivotal role in the regulation of skeletal muscle metabolism and the response to anti-oxidative stress, we examined the influence of regular exercise in human *BRCA* mutation carriers on their BRCA1 gene/protein expression and inflammatory/oxidative response. Sixteen *BRCA*-mutation carriers were assigned to an intervention (IG) or control group (CG). IG received a combination of high-intensity interval endurance (HIT) and strength training (HIRT) for six weeks, whereas CG received a low-intensity activity program. Before (T0) and at the end of the intervention (T1), muscle biopsy, physiological performance, blood withdrawal and anthropometry were obtained. Parameters included: Muscle BRCA1 gene/protein expression, inflammatory/oxidative stress, anti-oxidative capacity, peak oxygen capacity (VO_2_peak) and 1-repetition maximum (1-RM) at six different training machines. VO_2_peak and 1-RM of IG were increased at T1 compared to T0, whereas CG performance, physiological and molecular parameters remained unchanged. IG showed increased BRCA1 protein concentration as well as anti-oxidative capacity, whereas gene expression was unaltered. IG inflammatory and oxidative damage did not differ between time points. Combined HIT/HIRT increases aerobic and strength performance of *BRCA*-mutation carriers with up regulated BRCA1 protein expression and improved anti-oxidative status without showing an increased inflammatory response.

## 1. Introduction

Genetic predispositions account for the onset of 25–40% of all breast cancers before the age of 35 years [1]. Especially mutations in the BReast CAncer genes *BRCA1* and *BRCA2*, which are key genes for the regulation and repair of DNA, increase the risks for breast cancer to 55–60% and for ovarian cancer to 16–59% by age 70 years in women [2,3]. Women with a hereditary mutation in the BRCA-genes develop cancer approximately 20 years earlier than women without this gene mutation [4].

So far, there is not a single “golden standard” prevention method to reduce the risk of developing breast cancer in a *BRCA*-mutation carrier except prophylactic bilateral mastectomy or Salpingo-oophorectomy [5].

To avoid or minimize invasive surgical procedures, reducing the risk through regularly exercising may be an alternative. In addition to DNA repair and regulation, BRCA1 plays a pivotal role in the regulation of skeletal muscle metabolism [6] and is involved in the response to anti-oxidative stress [7]. While it is a well-established fact that regular exercise delays the onset of cancer in the general population as well as in breast cancer patients [8,9], and is therefore progressively used as primary and tertiary cancer therapy, there is a lack of actual training studies and their impact on metabolic and genetic profiles in BRCA gene mutation carriers.

So far only a few studies in rodents have shown an improved expression profile of *BRCA1* and an ameliorate energy metabolism by regular training, whereas heterologous *BRCA1* mutations have led to mitochondrial dysfunction, inhibition of lipolysis and subsequent lipid accumulation in the skeletal muscle [6,10]. Furthermore, BRCA1 up-regulation increases expression of the tumor suppressor gene p53 [4,11] highlighting the tight interaction between these pivotal regulatory contributors for genomic stability and normal cell proliferation. Studies have also shown that overweight associated with Type2-Diabetes and its secondary disease Cardiovascular disease (CVD) increases oncogenesis in *BRCA* mutation carriers [12,13,14]. Therefore, possibly counteracting oncogenesis by endurance and/or strength training might be a logical consequence. The positive impact of a structured, individualized endurance training in combination with nutrition education (based on the Mediterranean diet) was also recently published in a feasibility study [15].

Our group previously studied the effects of a six-week long combined endurance and strength training intervention in breast cancer survivors and found an increase in aerobic and strength capacity as well as reportedly higher life-quality [16]. The aim of this study was thus to determine the effect of the same training intervention in women and men with *BRCA* mutations on molecular and physiological parameters, including BRCA mRNA and protein expression, oxidative stress markers, inflammation markers, physiological performance and psychological well-being.

## 2. Results

### 2.1. Questionnaires

Life Orientation Test Revised (LOT-R) analyses revealed no change in the general optimism (*p* = 0.516) or pessimism (*p* = 0.174) in intervention group (IG) or control group (CG) (*p* = 0.224; *p* = 0.555), respectively. Anxiety (IG: *p* = 0.163; CG *p* = 0.141) and depression scales (IG *p* = 0.915; CG *p* = 0.110) determined by Hospital Anxiety and Depression Scale (HADS) remained unchanged between T0 and T1 for both groups. Statistical evaluation of group effect size showed small effect size (ES) of d = 0.30 for pessimism without any effect for optimism (ES d = 0.03), anxiety (ES d = 0.03) or depression (ES d = 0.11).

Compliance to complete the questionnaires and adherence to the training program was high.

### 2.2. Performance Parameters and Anthropometry 

**Figure 1:**
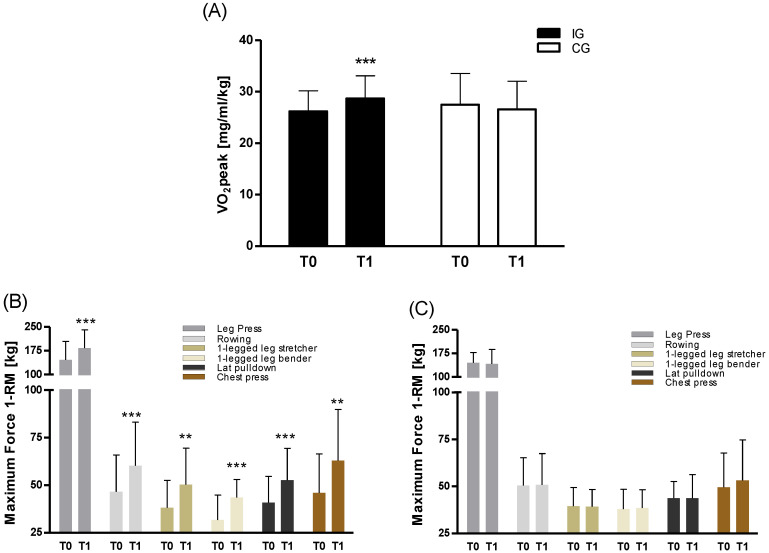
Performance parameters (**A**) VO_2_peak and 1-repetition maximum (1-RM) of (**B**) intervention group (IG) and (**C**) control group (CG) to evaluate changes in endurance and strength capacity, respectively. While there was a significant increase in VO_2_peak (*p* = 0.001) with high-intensity interval endurance (HIT) and in 1-RM at all six different training machines (*p*_mean_ = 0.007) with high intensity resistance training (HIRT) of IG (*n* = 10) at T1 compared to T0, CG (*n* = 6) performance parameters remained unchanged. A large time*group effect was observed for VO_2_peak with effect size (ES) d = 0.988 and 1-RM with ES_mean_ d = 0.870. ** *p* ≤ 0.01, *** *p* ≤ 0.001.

Peak oxygen consumption VO_2_peak of IG increased significantly (*p* = 0.001) from 26.2 ± 4.0 to 28.7 ± 4.4 mL/min/kg after the exercise intervention (Figure 1A). Mean strength performance, represented by 1-RM maximum determined at six different strength training machines, increased with an average significance level of *p*_mean_ = 0.007 (Figure 1B). Low intensity activity (LIA) performed by CG did not lead to any differences in endurance or strength capacity at T1 compared to T0. 

Except a minor group x time change in BMI and body weight, no difference was observed regarding basic anthropometric data in both groups over the study period (S1).

### 2.3. BRCA1 Protein and BRCA1 Gene Expression

**Figure 2:**
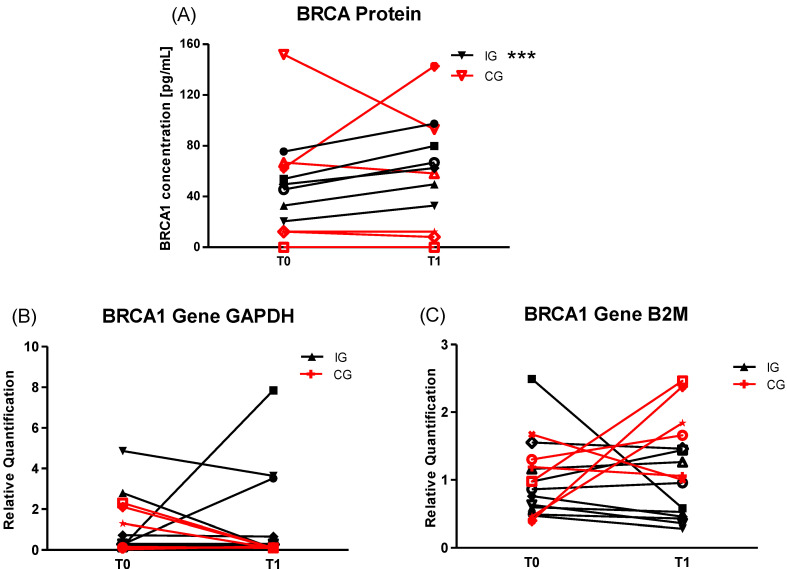
Protein concentration and gene expression of breast cancer (BRCA)1 of IG and CG after a 6-week long training intervention. (**A**) BRCA1 protein concentration of IG (*n* = 6) increased after the HIT/HIRT training (*p* < 0.001), whereas CG (*n* = 4) concentration remained unchanged and a small group*time effect (ES d = 0.295) was observed. Gene expression determined with *GAPDH* (**B**) and *B2M* (**C**) as respective reference gene did not show any significant changes in IG (*n* = 9) or CG (*n* = 6) T1 compared to T0, but a small group*time effect for *GAPDH* (ES d = 0.352) and a medium group*time effect for *B2M* (ES d = 0.600) were observed. *** *p* ≤ 0.001.

While IG *BRCA1* gene expression did not change either with *B2M* or *GAPDH* as a respective reference gene, BRCA1 protein concentration significantly increased in IG (*p* < 0.001) from 46.32 ± 18.78 to 64.83 ± 22.53 pg/mL (*p* < 0.001) with small time*group ES of d = 0.3. No changes were observed in CG in both BRCA1 protein and *BRCA1* gene expression (Figure 2). 

### 2.4. Immunology and Growth Factors

Results of analyzed immunological parameters and growth factors are summarized in Table 1. Neither the combined HIT/HIRT nor LIA induced any detectable inflammatory response in either treatment group. Analyzed cytokine concentrations did not differ between T0 and T1. The same was observed for growth factors IGF-1 and IGFBP-3.

### 2.5. Anti-Oxidative Status

**Figure 3:**
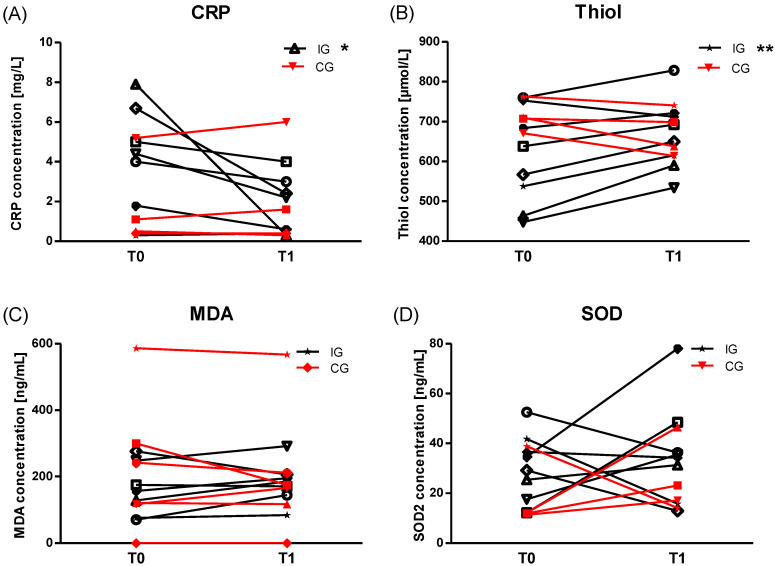
Plasma concentration of markers for anti-oxidative capacity and oxidative stress, respectively. (**A**) High-sensitive C-reactive protein (hsCRP) significantly (*p* = 0.05) decreased in IG (*n* = 7) at T1, whereas hsCRP concentration in CG (*n* = 4) remained unaltered (*p* = 0.32). (**B**) Total Thiol concentration increased in IG (*n* = 8, *p* = 0.009) but not in CG. For (**C**) MDA (IG *n* = 7, CG *n* = 6) and (**D**) SOD (IG *n* = 8, CG *n* = 4), no significant changes were observed in all groups, although a small group*time effect was seen for MDA (ES d = 0.44). * *p* ≤ 0.05, ** *p* ≤ 0.01.

C-reactive protein (CRP) concentration significantly decreased in IG (Figure 3A) with group*time ES d = 0.67, whereas total plasma Thiol concentration increased ((Figure 3B), *p* = 0.009, ES d = 1.2). Different reactions were seen in CG where both parameters remained unchanged (Figure 3A,B). Malondialdehyde (MDA) (Figure 3C) as well as Cu/Zn-Superoxid-Dismutase (SOD) (Figure 3D) did not differ between T0 and T1 in both groups. Unpublished data of our group of healthy non-*BRCA*-mutation carriers, who completed the same training protocol as IG over six weeks, revealed a comparable impact on total Thiol levels and unchanged SOD concentration. 

## 3. Discussion

Since the beginning of the 21st century, accumulating research has continued to show the positive effects of physical activity on cancer survivorship, so that the American Cancer Society now recommends a physical and nutritional healthy lifestyle to decrease the risk for tumor development and progression [17]. The inverse proportional connection between cardiorespiratory fitness and cancer mortality was evidenced in several studies [18,19], and recent studies estimate that successful lifestyle changes could prevent 25% to 30% of cases of breast cancer [20]. Findings in a study done by Pettapiece-Phillips et al. [21] indicate a direct increase of functional active *BCRA1* mRNA after a mainly sedentary lifestyle was ceased [21]. Furthermore, as BRCA1 is regarded as a regulator of skeletal muscle metabolism [6], a possible alternative or addition to current invasive therapy regimes may be the addition of regularly performed exercise and physiological training.

As, to the best of our knowledge, no study has so far examined the impact of a combined high intensity endurance and strength training intervention on the genetic, inflammatory, physiological and psychological profile in *BRCA1* mutation carriers, we aimed to examine these parameters in female and male *BRCA* mutation carriers before and after a six-week long HIRT and HIT training. The four main outcomes were (1) improved endurance and strength performance, (2) an increased BRCA1 protein expression with concurrent unchanged *BRCA1* expression, (3) a decreased CRP concentration with subsequently increased Thiol status and (4) an unchanged inflammatory status during the intervention. 

Type, timing and intensity of exercise in the treatment and prevention of cancer is a fundamental and controversial research topic in the field of sports medicine. Pijpe et al. (2010) showed that the intensity for individuals under the age of 30 is more important than the training volume [22], whereas data determined from a study by Nkondjock et al. (2006) did not exhibit any breast cancer risk reduction before diagnosis with regular moderate or intensive exercise [23]. On the other hand, other findings indicate that regular exercise and lack of obesity during adolescence decrease breast cancer risk in rodents and humans [24,25], delay the onset of breast cancer in women by approximately 10 years [4] and reduce the premenopausal breast cancer risk among *BRCA* mutation carriers [26]. These studies underline the current lack of understanding in the molecular mechanisms which are involved in the interaction of physical exercise, obesity, general life-style and breast cancer onset in *BRCA* mutation carriers. 

The positive and fast physiological adaptations of different types of high intensity training are well known and have been assessed for several stages in different diseases [27,28], including a combined HIT/HIRT training in breast cancer patients [16,29], but has not yet been studied in *BRCA* mutation carriers. We found the combined HIRT/HIT training resulted in a significant improvement of strength and aerobic capacity, despite no change in overall mental well-being could be observed, neither with HADS nor with LOT-R. To maintain a good physical condition in *BRCA*-mutation carriers is of high importance, since women with a *BRCA1* or *BRCA2* mutation face a 2-fold increase in the risk of diabetes 15 years after the diagnosis of breast cancer, which is further exacerbated by a high BMI [14]. To diminish these risk factors, endurance and strength training are useful systemic tools: a combined aerobic and resistance exercise program conducted over sixteen weeks in ethnically-diverse overweight or obese breast cancer survivors resulted in a significant reduction of fatigue and depression as well as beneficial adaptations in endurance, strength and bone health parameters [30].

As mentioned above, regular exercise training of rodents resulted in an improved expression profile of *BRCA1* and an improved energy metabolism, whereas the *BRCA1* mutation per se contributes to mitochondrial dysfunction, inhibition of lipolysis and subsequent lipid accumulation in the skeletal muscle [6,10]. Thus, we intended to analyse if any exercise-triggered upregulation of uncompromised BRCA1 in skeletal muscle of mutation carriers would modify the total amount of functional *BRCA1*.

Our exercise intervention up regulated the BRCA protein concentration in the intervention group, whereas the gene transcript remained unchanged. A possible explanation, apart from the fact of a small sample size or imperfect detection window, might be that training decreased the protein degradation by strengthening of the anti-oxidative system. This assumption is supported by increased serum Thiol concentration with concomitant decreased CRP concentration in IG. Thiol groups are one important component of the anti-oxidative defense system in human blood as they are able to react with alcoxy (RO*) and hydroxyl radicals (OH*) as well as to form disulphide bonds with harmful oxidized thiyl radicals and subsequently inactivate them [31]. Thus, Thiols are partly capable to reduce the progression of malignant oxidative processes on tissues and consequently act as a possible marker for the probability of breast cancer development [31]. In addition, high Thiol levels were shown to be beneficial for disease prognosis, whereas levels below reference values are associated with chronic diseases like heart insufficiency [32]. HIT training was shown to induce acute high oxidative stress in healthy and non-healthy individuals [33,34], but even after three weeks of training beneficial adaptations in the anti-oxidative defense were still seen [35,36], confirming our results. Noteworthy, our adaptions in the serum Thiol levels and BRCA1 protein expression of IG are similar to exercise-induced changes in healthy young men without *BRCA* mutation history, which executed a similar training program in our laboratory beforehand (unpublished results).

In addition to the beneficial increase in Thiol levels and strengthening of the anti-oxidative defense, decreased CRP concentrations after the six-week long training showed positive adaptations in the inflammatory response system. CRP is an acute-phase reactant inflammatory and established independent diagnostic marker for breast cancer development, as it is synthesized in hepatocytes in response to leucocyte-released cytokines within a tumor microenvironment [37]. Aggressiveness of the breast tumor can be estimated by the relative increase of CRP levels at the time of diagnosis [37]. In addition, the well-established fact that chronic inflammation can be a main contributor in the development and progression of carcinogenesis [38], the decreased CRP level found in our study after the HIT/HIRT underlines the importance to maintain a good training status in order to reduce harmful inflammatory responses. Although the cytokines tested and presented like IL-6 or IL-10 did not exhibit any change, CRP as an acute-phase reactant might show faster adaptations to the high intensity training than cytokines. As there is the same observed for the anti-oxidative enzyme SOD, a change in anti-/proinflammatory cytokines only after prolonged training can be assumed where six weeks of training might be too short. Published and comparable studies are scarce and show different results: Gonçalves et al. showed in their systematic review that evidence indicates that acute and chronic interventions may modify most immune markers, but aspects such as gender, contraceptive pill use in women, physical capacity of the investigated individuals, environment and type and intensity of the exercises may interfere with these markers as well as the data analysis [39]. Whereas acute exercise generally leads to an elevation of CRP [40] as well as IL-6 and some other cytokines, results of chronic exercise are inconsistent [41]. Whereas CRP concentration decreases regardless of age or sex of the individual and is associated with a decrease in BMI or %Fat [42], cytokine concentration seem not to alter in a similar degree and depend on a large scale on other factors like health status, age or body composition [39]. We therefore suggest that HIT/HIRT in our study may be appropriate even for untrained female and male individuals regardless of their age, as no change or increase in inflammatory parameters was observed prior to and post training.

With regards to the SOD protein analyte, it is not surprising that we had not detected any changes after the training intervention had concluded. The cytoplasmic Cu-/Zn-SOD binds to superoxide byproducts of oxidative phosphorylation, and mutations in this gene have been associated with premature aging and cancer confirming the link between the etiology of breast cancer and oxidative stress/lipid peroxidation [43]. In the last few years, different effects of exercise on SOD protein concentrations were found, depending on the exercise duration (acute or regular) and the SOD isoform, with unchanged or slightly increased levels [44]. With regards to HIIT, studies with rodents confirmed unchanged SOD expression with prolonged training [45], but studies in humans are still lacking. Thus, the long-term influence of HIT/HIRT on the different SOD-isoforms in humans has still to be determined.

In addition to unchanged SOD levels in all tested groups, lipid peroxidation, represented by MDA concentration, did not exhibit any change. 

This suggests the suitability of our training for beneficial molecular adaptations, since participants could cope with increased metabolic rates and its oxygen demands, because no negative impact on excessive superoxide formation, as experienced during acute high-intensity training [46], could be observed. The high demands of the high-intensity training led to an improved cardiopulmonal fitness and maximum strength in a short time. The resulting increased oxygen demand and increased metabolic rate could be important steps towards the development of suitable metabolic processes in the prevention of carcinogenesis in *BRCA* mutation carriers.

### Limitations

This study has several strengths and limitations. Especially the gain in aerobic capacity and strength as well as a high compliance underlines the feasibility of the performed HIT/HIRT. Nevertheless, the low participant number of *n* = 10 in IG and *n* = 6 in CG as well as the inclusion of both *BRCA1* and *BRCA2* gene mutation carriers might bias the statistical analysis. Therefore, we tried to minimize premature conclusions by effect size calculation and graphic representation of individual response. This study showed an increase in BRCA1 protein expression, but we did not test the functional efficiency of this protein. Hence, we can only speculate that the increased expression might exert beneficial effects in *BRCA1* and *BRCA2* gene mutation carriers, and that the increased expression did not led to the simultaneous increase of damaged proteins. To confirm the shown beneficial effect of the six-week long HIT/HIRT on the physiological level (aerobic capacity and strength) also on the molecular level, further studies with higher participant numbers and maybe an extended training period have to be conducted to improve the analyses of this pilot study.

## 4. Materials and Methods

### 4.1. Study Group

Nineteen participants (16w, 3m) were included in this prospective, randomized-controlled clinical study. Of those, 16 successfully completed the training. Inclusion criteria included proof of *BRCA1* and/or *BRCA2*-gene mutation, age between 18–69 years and a Karnofsky-index of at least 40%. All participants with present therapy of malignoma or comorbidities/factors which impeded training participation, were excluded. Both study groups were instructed to limit moderate physical activity to two hours per week in addition to the guided training intervention. A detailed statistical analysis of anthropometry data BMI [kg/m^2^], body weight [kg] and waist-to-height ratio is found in Table S1. Basal anthropometric parameters and health status of participants are summarized in Table 2 as well as in detailed form in Table S2. 

This study was registered in the German Register for Clinical Studies with the ID: DRKS00011410. The protocols used in this study were approved by the ethics committee (no. 141/14) of Ulm University and align with the Declaration of Helsinki. All participants gave written informed consent to participate in this study.

### 4.2. Assignment of Study Group and Training

**Figure 4:**
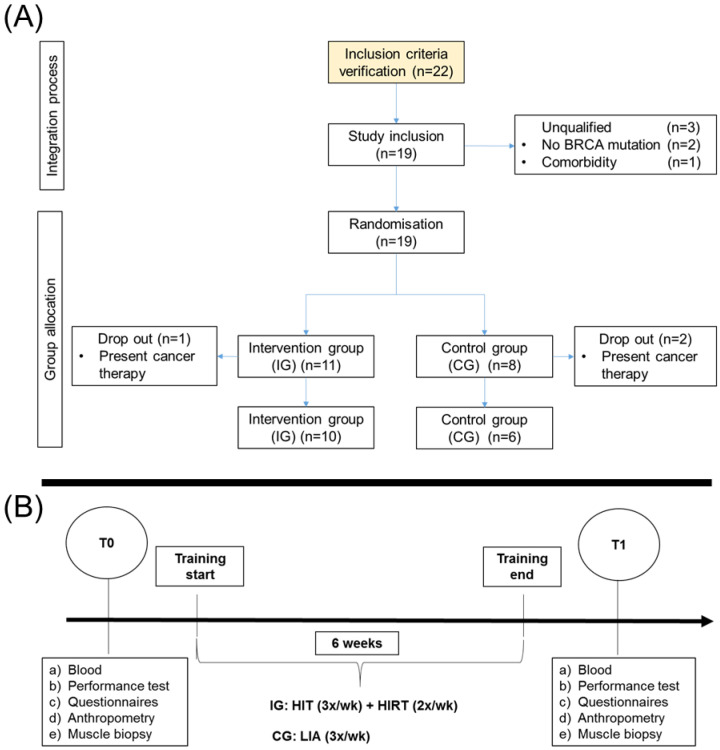
Description of randomization and study design. (**A**) Recruitment and assignment to the respective intervention (IG) or control group (CG) and the training setting performed by each group. Due to medical decisions and exclusion criteria, 16 out of 22 recruited participants completed the study successfully. (**B**) Study timeline with data collection time points and methods determined before (T0) and after (T1) a six-week training intervention of either combined high-intensity interval endurance (HIT) and strength training (HIRT) or a low intensity activity (LIA) for IG and CG.

Participants were assigned either to a control group (CG) or an intervention group (IG). A detailed description of the randomization process and the training timeline with data collection time points is shown in Figure 4. 

Participants in IG performed a six-week training period consisting of a combination of high-intensity interval training (HIT) and high intensity resistance training (HIRT), whereas CG performed low intensity activity (LIA). Data collection time points for parameter determination were at training start (T0) and three days after completion of the training at maximum (T1). 

In detail, the training program of IG included three trainings per week under professional supervision with at least one day of rest between training sessions. A combination of HIT and HIRT was performed on two days, whereas the training on one day consisted of HIT only. HIT was performed on a cycle ergometer (Lode^®^ Corival, Groningen, The Netherlands). The protocol was designed and monitored as in Schulz et al. [16]: 10 repetitions of one-minute peak loads at 85–100% of VO_2_peak, separated by one-minute intervals with reduced load. Before the intervals participants warmed up for a total of 10 min, and then completed 20 min of HIT followed by three minutes of cool-down. Warm-up and cool-down were performed at 50% of VO_2_peak. Subjective exhaustion, lactate, blood pressure and heart frequency were determined. By using lactate sampling, intensity of HIT was adjusted appropriately during the six-week period (target of Received Perception of Exertion 12–14; [48]).

A bicycle cardiopulmonary exercise test was completed according to international standards to determine VO_2_peak for HIT training load [49]. A step protocol (start at 25 W, increase 25 W every 3 min) was chosen for parallel lactate diagnostics [50]; the average oxygen uptake of the last 30 s before test abortion was defined as the VO_2_peak.

HIRT was performed at six different strength training machines (Leg Press, Leg extension, Leg Curl, Bench press, Rowing machine, Lat Pulldown (Technogym^®^, Cesena, Italy)). The one-repetition-maximum (1-RM), defined as the load that could be correctly executed once, was determined at T0 to evaluate the maximum strength of each participant [50] and the respective corresponding training load of 80% of the 1-RM. At each training machine, 2 sets of 8–12 exhausting 1-RM were performed with motion sequence (concentric 2 sec, isometric 1 sec, eccentric 4 sec) and adjusted every two weeks where individual training loads appeared to be too low after strength gains. To increase the intensity of the strength training, the number of repetitions (up to 12) was increased prior to weight adjustments if necessary [51]. Resting time between different sets was three minutes.

LIA of CG included relaxation exercises like Yoga three times per week without any resistance or endurance strain. Time of professional supervision for IG and CG was evenly distributed. 

### 4.3. Psychological Parameters/ Questionnaires

Grade of optimism was determined with the Life Orientation Test Revised (LOT-R) developed by Scheier et al. [52] which supports the distinction between generalized optimism and pessimism, and is suitable for individual and group analysis. In addition, the Hospital Anxiety and Depression Scale (HADS) was used, which is an established tool for the determination of anxiety and depression of adults with physical disabilities [53,54].

### 4.4. Muscle Biopsy and Analysis

Muscle biopsy was conducted at T0 and T1 employing the Bergström technique according to published protocols [55] to gain a maximum of approximately 200 mg muscle tissue from the *musculus vastus lateralis*. For RNA isolation, muscle tissue was incubated for 24 h with RNAlater (QIAGEN GmbH, Hilden, Germany) at 4 °C and then stored in cryotubes at −80 °C until further analysis. For protein examination, muscle tissue was immediately cryopreserved with liquid nitrogen and stored at −80 °C until further analysis.

### 4.5. RNA Isolation and BRCA1 Analysis

Muscle tissue RNA was isolated with the RNeasy^®^ Plus Mini Kit (QIAGEN GmbH, Hilden, Germany) according to the manufacturer’s instructions. RNA was quantified with spectrophotometry (NanoDrop 2000c, Thermo Scientific, Massachusetts, MA, United States) and transcribed to cDNA with the QuantiTect^®^ Reverse Transcription Kit (QIAGEN GmbH, Hilden, Germany) according to the manufacturer’s instructions. cDNA was used to determine the expression of *BRCA1* with Real Time PCR (RT-PCR) analogous to established protocols [56]. Since the choice of reference gene can impact the outcome, *GAPDH* and *B2M* were used as established reference genes for either endurance or combined endurance/strength exercise, respectively [56,57].

#### BRCA1 Protein Determination

BRCA1 protein concentration from venous serum was analyzed with the Human BRCA1 ELISA (Abbexa Ltd., Cambridge, UK) according to manufacturer’s instructions. 

### 4.6. Growth Factors and Immunology

Concentration of growth factors IGF-1 and IGFBP-3 was determined with the IDS-iSYS Multi-Discipline Automated System (Immunodiagnostic Systems Holdings, Boldon Colliery, United Kingdom). Immunological parameters Interleukine-2 receptor (IL2r), Interleukine-1ß (IL1ß), Interleukine-10 (IL10), Tumor-Necrosis-Factor-α (TNF-α) were determined by chemiluminescence with the Immulite 1000 (Siemens Healthcare GmbH, Erlangen, Germany) and Interleukine-6 (IL6) and Growth/differentiation factor 15 (GDF15) were measured by ElectroChemiLuminescenceImmunoAssay (ECLIA) with the Cobas 8000 (Roche, Basel, Switzerland).

### 4.7. Oxidative Stress Markers and Anti-Oxidative Capacity

MDA (Elabscience; E-EL-0060, Wuhan, China), hsCRP (IBL international; EU59151, Männedorf, Switzerland), total Thiol (Immundiagnostik; K1800, Bensheim, Germany) and SOD (Immundiagnostik; K7120, Bensheim, Germany) protein concentrations from venous serum were analyzed using commercial ELISAs according to the manufacturers’ instructions.

### 4.8. Statistical Analysis

Statistical analysis was performed using the SPSS software package (SPSS Statistics 21.0, https://www.ibm.com/, IBM, Ehningen, Germany) and GraphPad PRISM (Version 6.04, https://www.graphpad.com/scientific-software/prism/, San Diego, CA, USA). Data were tested for Gaussian distribution and group differences were tested using by 1-way ANOVA with repeated measures and two-sided t-test to determine statistical significance and effect size (ES). Descriptive statistics of the data was presented as mean ± standard deviation. Statistical differences were considered to be significant for values of * *p* ≤ 0.05, ** *p* ≤ 0.01, *** *p* ≤ 0.001.

ES was calculated after 1-way ANOVA with d ≥ 0.2 (small effect), d ≥ 0.5 (medium effect) and d ≥ 0.8 (large effect) according to Cohen’s d [58].

Questionnaires LOT-R and HADS were analyzed with reference to established protocols of Glaesmer et al. and Herrmann and Buss [59,60].

## 5. Conclusions

This pilot study with 16 *BRCA*-mutation carriers which are prone to cancer onset showed, that a combined HIT/HIRT training increased physical performance of this study population with positive effects on BRCA1 protein expressions as well as the systemic anti-oxidative status, while no proinflammatory response was seen. The high adherence and compliance to the training program supports the importance of suitable and regular high intensity training in the daily life of individuals with an increased risk of breast cancer. To evaluate the necessary detailed mechanism on the molecular level and to support subjects with an increased genetic risk for developing breast cancer, further studies with higher numbers of participants are needed to implement HIT/HIRT program as a scientifically sound prospective prevention and/or treatment method to reduce the risk for the development of cancer in individuals with *BRCA1* mutations.

## Figures and Tables

**Table 1:** Immunological parameters T0 and T1 of IG and CG with respective *p*-value (Mean ± SD).

Parameter	Group	T0	T1	*p*-Value
IGF-1 [μg/L]	CG (*n* = 6)	121.5 ± 71.7	104.3 ± 38.3	0.379
	IG (*n* = 9)	181.4 ± 72.7	207.4 ± 51.3	0.450
IGFBP-3 [μg/dL]	CG (*n* = 6)	93.5 ± 28.5	89.2 ± 24.6	0.465
	IG (*n* = 10)	70.7 ± 21.8	88.8 ± 17.6	0.063
IL-2R [U/mL]	CG (*n* = 5)	414.2 ± 215.4	353.2 ± 98.3	0.540
	IG (*n* = 9)	379.8 ± 145.9	471.1 ± 158.2	0.155
GDF-15 [pg/mL]	CG (*n* = 6)	467.1 ± 187.0	511.3 ± 174.4	0.485
	IG (*n* = 10)	391.2 ± 213.1	383.3 ± 161.3	0.872
TNF-α [pg/mL]	CG (*n* = 5)	5.4 ± 1.6	6.3 ± 3.4	0.664
	IG (*n* = 9)	5.9 ± 2.6	6.9 ± 2.7	0.191
IL-6 [pg/mL]	CG (*n* = 5)	2.0 ± 1.1	1.9 ± 0.6	0.672
	IG (*n* = 9)	1.6 ± 0.4	1.6 ± 0.3	0.351
IL-1β [pg/mL]	CG (*n* = 5)	2.9 ± 1.9	2.9 ± 1.9	1.000
	IG (*n* = 9)	2.3 ± 1.5	2.5 ± 1.7	0.356
IL-10 [pg/mL]	CG (*n* = 5)	3.6 ± 2.5	2.9 ± 2.0	0.374
	IG (*n* = 10)	2.0 ± 1.7	1.9 ± 1.8	0.347

**Table 2:** Basal anthropometric parameters, current health status and activity level of IG and CG.

Parameter	IG (*n* = 10)		CG (*n* = 6)	
	Mean (SD)	CI (5;95)	Mean (SD)	CI (5;95)
Age (years) *^1^	35.5 (10.5)	(28.0; 43.0)	46.3 (5.3)	(40.8; 51.9)
Body size (cm) *^1^	170 (0.1)	(165; 174)	171 (0.1)	(161; 181)
Body mass (kg) *^1^	75.8 (14.1)	(65.7; 85.9)	72.0 (16.0)	(55.3; 88.7)
Waist-to-height ratio *^1^	0.51 (0.04)	(0.48; 0.53)	0.55 (0.13)	(0.41; 0.69)
Sex (men/women) *^2^	2/8		1/5	
BRCA (1/2) *^2^	5/5		3/3	
Menopausal status (pre/peri)	6/2		3/2	
*Medical treatment before study participation*				
Mastectomy/adenectomy	0/2		1/3	
Treated breast cancer (chemotherapy/irradiation)	2 (2/1)		2 (2/1)	
Ovarian cancer	0		0	
High daily physical activity *^3^	6		3	
Average of physically strenuous, sweat-inducing activities	1 time per week		2–4 times per week	

*^1^ unpaired Student’s t-test, *^2^ c^2^-test. *^3^ AAS ≥ 83 [47] / SD = standard deviation, CI = 95% confidence interval (lower limit; upper limit).

## Supplementary Materials

The following are available online at https://www.mdpi.com/2072-6694/12/6/1526/s1, Table S1: Detailed statistical analysis with intra-/intergroup differences over time of anthropometric parameters BMI, body weight and waist-to-height ratio of IG and CG, Table S2: Basal anthropometric parameters, current health status and activity level of IG and CG.
