# Supplementary material for: Beneficial Molecular Adaptations in BRCA-Mutation Carriers by Combined HIT/HIRT Intervention: Results from a Pilot Study
Source: Cancers (Basel). 2020 Jun 10;12(6):1526. doi: 10.3390/cancers12061526 (PMC7352264; doi:10.3390/cancers12061526)
Supplement: Supplementary file 1 [file cancers-12-01526-s001.pdf]

Supplemental Table 1: Detailed statistical analysis with intra-/intergroup differences over time of anthropometric parameters BMI, body weight and waist-to-height ratio of IG and CG.

	IG (n=10)						CG (n=6)								
	Pre		Post		Change (%)	Pre vs. post	Pre		Post		Change (%)	Pre vs. post	Group	Time	Group x time
Parameter	mean (SD)	CI (5;95)	mean (SD)	CI (5;95)	mean (SD)	p-value[a]	mean (SD)	CI (5;95)	mean (SD)	CI (5;95)	mean (SD)	p-value[a]	p-value[b]	p-value[b]	p-value[b]
Anthropometry															
BMI[c] [kg/m²]	26.4 (4.9)	(22.8; 30.0)	26.2 (5.1)	(22.4; 30.0)	0.8 (2.3)	0.358	24.7 (6.1)	(20.0; 29.3)	25.1 (6.4)	(20.2; 30.0)	1.5 (1.3)	0.062	0.625	0.417	0.050
Body weight [kg]	75.8 (14.1)	(65.8; 85.8)	75.3 (17.8)	(64.7; 85.9)	-0.8 (2.3)	0.370	72.0 (16.0)	(59.1; 84.9)	73.2 (17.0)	(59.5; 86.9)	1.5 (1.3)	0.058	0.710	0.373	0.046
Waist-to-height ratio	0.52 (0.09)	(0.46; 0.58)	0.51 (0.09)	(0.45; 0.57)	-1.3 (1.8)	0.066	0.53 (0.09)	(0.45; 0.61)	0.53 (0.09)	(0.45; 0.61)	0.04 (1.7)	1.000	0.785	0.227	0.227

SD = standard deviation, CI = 95% confidence interval (lower limit; upper limit).

[a] Paired samples *t*-test.

[b] Repeated measures ANOVA.

[c] Body-Mass-Index.

Supplemental Table 2: Basal anthropometric parameters, current health status and activity level of IG and CG.

	IG (n=10)		CG (n=6)		
	**Mean (SD)**	**CI (5;95)**	**Mean (SD)**	**CI (5;95)**	**p-value**
**Age (years)**	35.5 (10.5)	(28.0; 43.0)	46.3 (5.3)	(40.8; 51.9)	0.035[1]
**Body size (cm)**	170 (0.1)	(165; 174)	171 (0.1)	(161; 181)	0.681[1]
**Body weight (kg)**	75.8 (14.1)	(65.7; 85.9)	72.0 (16.0)	(55.3; 88.7)	0.625[1]
**Waist-to-height ratio**	0.51 (0.04)	(0.48; 0.53)	0.55 (0.13)	(0.41; 0.69)	0.838[1]
**Gender (men/women)**	2/8		1/5		0.869[2]
**BRCA (1/2)**	5/5		3/3		1.000[2]
**Menopausal status (pre/peri)**	6/2		3/2		
Medical treatment before study participation					
Mastectomy/adenectomy	0/2		1/3		
Treated breast cancer (chemotherapy/irradiation)	2 (2/1)		2 (2/1)		
Ovarian cancer	0		0		
**High daily physical activity[3]**	6		3		
**Average of physically strenuous, sweat-inducing activities**	1 time per week		2-4 times per week		

[1] Unpaired Student's t-test; [2] χ^2-test; [3] AAS ≥83 (1); SD = standard deviation;
CI = 95% confidence interval (lower limit, upper limit).

1 Daughton D, Fix A. Human activity profile: professional manual. Odessa, FL: Psychological Assessment Resources, Inc.; 1988.
